# Resource Utilization and Caring Cost of People Living with Human Immunodeficiency Virus (PLHIV) in Saudi Arabia: A Tertiary Care University Hospital Experience

**DOI:** 10.3390/healthcare10010118

**Published:** 2022-01-07

**Authors:** Mazin Barry, Leen Ghonem, Nourah Albeeshi, Maha Alrabiah, Aynaa Alsharidi, Hussain Abdulrahman Al-Omar

**Affiliations:** 1 Division of Infectious Diseases, Department of Internal Medicine, College of Medicine, King Saud University and King Saud University Medical City, Riyadh 11451, Saudi Arabia; mbarry@ksu.edu.sa (M.B.); aalsharidi@ksu.edu.sa (A.A.); 2 Department of Pharmacy, King Saud University Medical City, Riyadh 12372, Saudi Arabia; Leen_ghonem@hotmail.com; 3 Department of Neurosciences, King Faisal Specialist Hospital and Research Center, Riyadh 11564, Saudi Arabia; Nalbeeshi@kfshrc.edu.sa; 4 Department of Internal Medicine, College of Medicine, King Saud University and King Saud University Medical City, Riyadh 12372, Saudi Arabia; Maha.a.alrabiah@gmail.com; 5 Department of Clinical Pharmacy, College of Pharmacy, King Saud University, Riyadh 11451, Saudi Arabia; 6 Health Technology Assessment Unit, College of Pharmacy, King Saud University, Riyadh 11451, Saudi Arabia

**Keywords:** HIV, medical care, antiretroviral therapy, resource utilization, tertiary care hospital, Saudi Arabia

## Abstract

The human immunodeficiency virus (HIV) is associated with a significant burden of disease, including medical and non-medical costs. Therefore, it is considered to be a priority for all health authorities. The aim of this study is to determine healthcare and treatment costs of caring for PLHIV at one of the tertiary care university hospitals in Riyadh, Saudi Arabia. This was a micro-costing, retrospective, observational study from a tertiary care university hospital and included all confirmed HIV-infected patients who visited infectious disease clinics in the period from 1 January 2015 to 31 December 2018. A total of 42 PLHIV were included in this study. The mean age of the study participants was 38.76 ± 11.47 years with a mean disease duration of 5.27 ± 4.81 years. The majority of patients were male (85.7%) and Saudi (88.1%). More than half of included patients (59.5%) had a CD4 count of more than 500. During the study period, 26 patients (61.9%) were initiated on a single-tablet regimen. Overall, the main cost-driver was antiretroviral medications, which cumulatively represented more than 64% of the total cost. Patients who developed opportunistic infections had a statistically significant (*p* = 0.033) higher financial impact, both as a total and on a patient level, than those presented without opportunistic infections. On a patient level, the mean and median costs were higher and statistically significant for those with co-morbidities than those without co-morbidities (*p* = 0.002). The majority of the economic burden of PLHIV is attributable to antiretroviral therapy use. The healthcare costs of PLHIV can vary greatly, depending on the presenting illness, clinical stage, developed opportunistic infection, co-morbidity, and pharmacological therapy.

## 1. Introduction

The human immunodeficiency virus (HIV), the etiological agent of acquired immunodeficiency syndrome (AIDS), has led to a significant burden throughout the world, in terms of both on the medical and economical levels [1]. Despite the marked reduction in the number of new HIV infections, AIDS-related deaths, and major scientific advances in HIV therapies, serious challenges remain in addressing the global HIV pandemic and ultimately bringing it to an end [2].

According to the latest report issued by the Joint United Nations Programme on HIV/AIDS (UNAIDS), around 38 million people (36.2 million adults and 1.8 million children under 15 years of age) are living with HIV (PLHIV). Since the onset of the epidemic up to the end of 2020, there have been more than 34.7 million AIDS-related deaths [3]. The spread of HIV in the Middle East and North Africa (MENA) region has increased by 22% [4], although there is relatively low prevalence in Saudi Arabia. Up to December 2014, there were 21,761 reported cases of HIV among Saudis and non-Saudis [5]. By the end of 2018, the total reported cases of all diagnosed HIV infections among Saudi nationals were 8148, with 533 newly diagnosed cases during that year [6]. However, it is difficult to ascertain the true figures due to limited data availability [4].

Medical advances that have enabled earlier diagnosis of HIV and development of highly active antiretroviral therapy (ARTs) have increased the chances of PLHIV living for longer. However, these advances have also meant that medical approaches have needed to focus more on chronic disease management than on clinical management. Patients living longer with HIV means that there is a higher per capita cost compared to other chronic diseases; some patients survive decades of treatment, medication use, and healthcare visits. Further, there is also an increased risk of non-AIDS-related morbidity among PLHIV, which is placing even more strain on healthcare resources. Concerns are growing about the lifetime care costs of PLHIV, who not only need HIV medication for longer, but also frequently need treatment for co-morbid conditions [7].

A recent study from the United States (US) estimated the lifetime medical costs for a 35-year-old person infected with HIV to be $326,500, with 60% of those expenses attributable to the costs of ARTs [8]. Along similar lines, according to a study in France, treating patients with a CD4 cell count of less than 50 cells/mm^3^ costs around three times as much a year as treating those with less-compromised immune function, that is, a CD4 cell count of greater than 500 cells/mm^3^; the biggest drivers behind the extra costs were ARTs and inpatient hospitalizations [9]. Moreover, in the south of Ireland in 2012, a bottom-up costing study of routine HIV outpatient care performed in a regional referral center estimated the monthly cost per patient to be €973, with almost 90% being spent on ARTs [9].

In Saudi Arabia, resource utilization and the cost of healthcare associated with medical care for PLHIV is not yet available. Examining the HIV epidemic through an economic lens can enable healthcare payers and decision-makers to form better budgeting plans to serve the needs of their patients; facilitate assessment of the value of new HIV treatments and treatment strategies; and provide information about the cost of HIV that may augment the prioritization of research efforts and create a process of evidence-based policy development. Extrapolating findings from studies in different countries is generally inappropriate due to variations in how health services are provided and organized. Therefore, the purpose of this study was to determine the healthcare and treatment costs (direct medical costs) of caring for PLHIV at one of the tertiary care university hospitals in Riyadh, Saudi Arabia.

## 2. Materials and Methods

### 2.1. Study Design

This was a retrospective, observational study from a tertiary care university hospital. The study included all confirmed HIV-positive patients who visited King Saud University Medical City (KSUMC), a tertiary care university-based referral hospital with a bed capacity of 1200, in the period from 1 January 2015 to 31 December 2018. The study included all HIV-positive individuals aged 18 years and older who were diagnosed and followed up at KSUMC infectious disease clinics for more than a year. Patients with a negative P24 antigen confirmatory test or HIV-positive individuals who did not visit infectious disease clinics were excluded from the study.

### 2.2. Data Collection

Patients’ data were collected between 1 December 2020 and 31 March 2021. Patients’ consent was not required due to the nature and type of the study (non-interventional study). For each individual identified as an HIV patient, the following information was retrieved from the hospital health information system (HIS), which included data on patients’ characteristics: gender, age group, city of residence, employment status, education, symptoms at first health encounter, mode of diagnosis, mode of transmission, and co-morbidities. For the purposes of resource utilization calculations, we retrieved information on the dates and numbers of diagnostic imaging tests, laboratory tests, and received medications for each accident and emergency visit, outpatient department visit, and inpatient admission.

For laboratory tests, we retrieved the numbers of the following tests that were performed for included patients: the blood chemistry study, blood lipid profile, brain natriuretic peptide testing, complement, complete blood count, c-reactive protein test, prothrombin time test, erythrocyte sedimentation rate, liver function test, peripheral blood smear, rheumatoid factor, antinuclear antibody test, metabolic profile, urinalysis, kidney function test, HIV viral load, CD4 cell count and percentage, microbiology, and screening tests for opportunistic infections. Both genotyping and HLA-B5701 tests were excluded from our calculation as they were not routinely done in our hospital. For diagnostic imaging, we included X-rays, CT scans, magnetic resonance imaging, and ultrasounds, as well as procedures like endoscopy. For medications, we collected data related to HIV therapies only, including initial and current ARTs, doses, frequencies, starting dates, stopping dates, and total numbers of dispensed tablets.

The costs attributed to PLHIV care was estimated from the payer’s perspective. The unit costs of all healthcare services and ARTs for included patients were estimated by a detailed bottom-up (micro-costing) approach to arrive at the most precise cost estimates. In micro-costing, all resources used are identified, and then the unit costs of the resources are multiplied by the quantities identified [10]. In this study, each patient’s use of a service or therapy was multiplied by the unit cost for that particular service or therapy used over the whole study period and per year, whereas for the total costs per patient, we based our calculations on the sum of the visits per department, numbers of diagnostic tests, laboratory tests, and admissions, and hospitalization costs over the whole study period and per year.

For healthcare professionals, costings for those who are routinely involved in providing care for HIV patients (nurses, physicians, phlebotomists, pharmacists, and technicians) per service department were included. The costs of diagnostic procedures, laboratory tests, visits, and admissions were matched with the costs of services for the study’s Gregorian year. Medications unit costs were obtained from the hospital’s pharmacy department. All costing results were calculated in Saudi riyals and then converted to US dollars by dividing the resultant cost by 3.75 (fixed exchange rate).

### 2.3. Statistical Analysis

All collected information was entered into a Microsoft Excel^®^ spreadsheet for data sorting and cleaning before being exported to a statistical package software and coded for data analysis. Descriptive statistics were calculated for patients’ demographics and clinical characteristics using frequencies, median, the first quartile (Q1) and third quartile (Q3), mean, and standard deviation (SD). The statistical differences among patients’ characteristics groups and for resource utilization for ARTs, laboratory tests, hospital admission, and radiology services (the total costs from the whole study period for each healthcare service and ARTs per patients’ characteristics) were compared using the non-parametric Wilcoxon rank-sum test for groups’ medians due to the small sample size and non-normally distributed outcomes. Results of Wilcoxon rank-sum tests hypothesize that the median effects in the two groups being compared are equal. Statistical significance was defined by the two-sided test with a *p*-value < 0.05. All statistical calculations were performed using IBM^®^SPSS^®^ Version 24.0 (Armonk, NY, USA: IBM Corporation, 2016).

### 2.4. Ethical Approval

The study was conducted according to the guidelines of the Declaration of Helsinki, and approved by the King Saud University’s College of Medicine Research Ethics Committee (IRB: E-18-3309).

## 3. Results

### 3.1. Patients’ Characteristics

In total, 67 patients were identified as HIV patients, and 42 of them met our inclusion criteria. The mean age of the participants was 38.76 ± 11.47 years, with a mean age at diagnosis of 33.73 ± 11.63 and a mean disease duration of 5.27 ± 4.81 years. The majority of patients was male (85.7%) and Saudi (88.1%). Fewer than two-thirds of the included PLHIV were married, and 50% of the total participants were employed. The sociodemographics of the included PLHIV are shown in Table 1.

More than half of the included patients (59.5%) had a CD4 count of more than 500. Additionally, 69% of patients refused to disclose the mode of their HIV transmission, and 26% reported sexual intercourse as the main reason for their acquiring HIV. More than two-thirds of patients presented without co-morbidities, and 50% of the patients first presented or developed at least one opportunistic infection during the study period; the most frequently reported opportunistic infection was Pneumocystis jirovecii pneumonia (18.9%). During the study period, 26 patients (61.9%) were initiated on a single-tablet regimen (STR) consisting of either (Elvitegravir/Cobicistat/Emtricitabine/Tenofovir Disoproxil Fumarate) or (Efavirenz/Tenofovir Disoproxil Fumarate/Emtricitabine). Of the total number of patients who received a multi-tablet regimen (MTR), only three subjects continued on such regimens. Table 2 describes the clinical characteristics and treatment regimens of the PLHIV in this study.

### 3.2. Healthcare Resource Utilization and Costs

The financial impact (as a whole cost and as a percentage of the total cost) of each cost category is reported in Table 3. The total cost to provide healthcare to an HIV-infected patient increased by 56% from US$200,595.74 in 2015 to US$455,032.89 in 2016, then by 32% to US$668,852.10 in 2017, driven by the cost of hospital admission to medical wards and intensive care units. It then dropped by 28% to US$479,909.72 in 2018. Overall, the main cost driver was ARTs, which cumulatively represented more than 64% of the total cost. This cost category constantly increased, both as a whole cost and as a percentage trend during the study period, from US$125,494.94 in 2015 to US$390,828.99 in 2018 (62.56% in 2015 to 81.44% in 2018) (Table 3).

Total expenditure and breakdowns according to patients’ demographics and clinical characteristics are presented in Table 4. Male patients had a higher total impact on cost than female patients, with total costs of US$1,531,478.13 and US$272,912.27, respectively. In comparison with female patients, total costs were 69.8% higher for male patients. On a patient level, female patients had a higher average cost compared with male patients. Patients who developed opportunistic infections had a statistically significant (*p* = 0.033) higher financial impact, both as a total and on a patient level, than those who presented without infection: the total estimated costs were US$1,108,900.27 and US$695,490.40, respectively. Costs were more than six times higher for hospital admission and three times higher for radiology; costs of laboratory tests doubled for patients who developed infection. The cost impact of patients with co-morbidities was lower than that of patients without co-morbidities as a total, the figures being US$876,531.73 and US$927,858.67, respectively. However, on a patient level, the mean and median of costs were higher and statistically significant for those with co-morbidities than those without (*p* = 0.002) (Table 4).

## 4. Discussion

Worldwide, countries are confronted with economic constraints, and resources are becoming even scarcer, thus the need to optimize the use of healthcare resources based on the population’s needs. Policy- and decision-makers rely heavily on costing information to allocate resources, so estimating the true cost of necessary healthcare as accurately as possible must be the first step in planning specialized health services. The present study appears to be the first attempt to quantify how medical resources are used and costed among PLHIV in Saudi Arabia. The total estimated cost of caring for those PLHIV included in this study over the study period was US$1,804,390.45, nearly 65% of which was allocated to ARTs. A prospective cohort study by Maheswaran et al. quantified the impact of HIV infection on numbers of adults admitted to medical wards, and found that HIV-related hospital care places substantial financial burdens on health systems and patients [11]. Recently, Ritchwood et al. reported similar findings to the present study, demonstrating that expenditures associated with HIV care over a 10-year period were significantly higher between 2002 and 2011 than for those not infected with HIV. The estimated total direct expenditure for HIV/AIDS care and treatment between 2002 and 2011 was US$10.7 billion [12].

In this study, HIV-positive patients were predominantly male (85.7%). In Saudi Arabia, the HIV epidemic is more common among males compared with females, with a male-to-female ratio of 4:1 among the newly detected cases [5]. However, this ratio could be subject to statistical bias, since men are more likely to be screened or tested, for instance, when they apply to work as a healthcare professional or enroll in military school or service. It is anticipated that the male-to-female ratio will decrease as more infected males infect their female sexual partners [13]. In Saudi Arabia, most women get infected with HIV through their husbands, who mostly were not aware of their HIV status; they rarely spread the infection to the rest of the population, apart from occasional vertical transmission to their own children [14]. This is similar to the results from other studies which reported higher infection rates in the male gender [15]. Surprisingly, half of our patients were employed, which was also reported by several other studies [16,17]. Employment in PLHIV can have positive outcomes on their social interactions, economic status, self-esteem, and mental health, as well as their country’s economy [18,19]. In Saudi Arabia, the government issued a law to ensure that Saudis infected with HIV have the right to sustain their work status and education. This can justify the 50% employment rate in our study. Globally, it has been found that PLHIV experiences unemployment rates three times higher than the average national unemployment rates [20]. Possible reasons for unemployment of PLHIV may include stigma, discrimination, restrictive policies and practices, and HIV-related illnesses [19]. However, this figure is changing due to the advancement in HIV treatment, particularly with the introduction of ARTs which have shown to have a positive impact on both job retention and the time needed to return to work in PLHIV [21,22].

In our study, a substantial proportion of the total medical cost was associated with ARTs; they accounted for more than 65% of total hospital spending, with costs of prescription medications increasing over time. Our results are consistent with the results from several studies from different parts of the world that have demonstrated the main cost driver among PLHIV to be ARTs [8,12,23,24,25,26,27,28,29]. Although they are expensive, ARTs are cost-effective [30,31] due to their capabilities in reducing viral load to almost undetectable levels and partially enabling immune restoration, thereby preventing the onset and recurrence of opportunistic infections while significantly reducing the probability of transmitting infection to others [32,33]. Since lifelong ARTs are needed for PLHIV, use of bioequivalent generic formulations as replacements for patients currently administering off-patented ARTs could potentially lower the costs of treatment and care [34]. Although switching to generics should be straightforward, consideration must be given to how patients and physicians might feel about it, whether switching might make patients less likely to adhere to their treatment regimen, and the availability and stability of the generic supply chain. Where clinically appropriate, it is likely that patients newly starting on ARTs could be offered a generic formulation of their off-patented branded therapies.

In this study, all the patients received combination ARTs in the form of three or more drugs or as a single-tablet or multi-tablet regimen. Use of combination ARTs has been shown to be very cost-effective [7,35,36] and associated with several positive outcomes, such as lowering inpatient medical care resources; this is because they can durably halt HIV disease progression, decrease the incidence of opportunistic infections and hospital admissions, and decrease lengths of stay and deaths [28]. They also have the important population-level benefit of reducing onward transmission [37]. However, they also prolong patients’ survival, and therefore will lead to increased total lifetime medical costs. Part of these increased costs is due to combination ARTs’ adverse effects and the increased incidence of complications from co-morbidities, frequently associated with HIV disease; another part of the increased costs is to do with more financial resources being needed for laboratories, pharmacies, and outpatient visits [7,28,35]. Additionally, the results demonstrated a direct proportional increase in the ARTs cost between 2015 and 2018; this cost constantly increased as an absolute value for all the years and as a percentage trend except in 2017, which was associated with a drop by 12%, compared with year 2016, in ARTs percentage of the total patient cost as a result of the remarkable increase in patients’ admission costs that year. The increase in the cost of HIV medications could be explained by the increased prescriptions and switching of patients from their initial, relatively cheaper, MTRs to more potent, tolerable, once-daily, fixed-dose combinations of efavirenz, emtricitabine, and tenofovir. Despite their high prices, fixed-dose combinations are considered cost-effective and even cost-saving [38,39] due to the lower need for hospitalization and indirect medical cost savings, better adherence, and better viral suppression [40,41]. They also have the important societal benefits of preventing the emergence of treatment resistance and minimizing the risk of HIV transmission [42].

In terms of spending on healthcare services, PLHIV with HIV and non-HIV co-morbidities or those who developed opportunistic infections including tuberculosis, candidiasis, cryptococcal meningitis, toxoplasma encephalopathy, and pneumonia had higher average resource utilization per service than those without co-morbidities or non-opportunistic infections, particularly for hospital admission and laboratory testing. It is documented that PLHIV has higher prevalence of all chronic diseases than the general population. In fact, non-infectious co-morbidities account for more than 50% of deaths in PLHIV, led by cardiovascular diseases and cancer [43]. Together, opportunistic infections and co-morbidities increase costs and require more healthcare resources. In this study, we noticed a gradual spike in hospital admission cost with a peak reached in 2017, followed by a drop in admission costs. This was driven by the admission of few PLHIVs in that year to the intensive care unit (ICU) to treat HIV-related co-morbidities and opportunistic infections. This result is similar to results from recent studies among PLHIV in France, Italy, and China which showed that most patients presenting to the hospital with an opportunistic infection or co-morbidity had increased hospitalization costs [9,29,43,44,45]. This evidence highlights the importance of proper initiation and tailoring of ARTs and management of co-morbidities among PLHIV in order to reduce such spending [29].

From policy and practice perspectives, since ARTs are the main driver for the high cost associated with PLHIV care and they can offer the greatest opportunity for cost savings, policy- and decision-makers should plan and target their strategies primarily toward medication-related initiatives. In addition to the use of generic formulation, simplification of regimens, development of a national treatment guideline, and engaging in managed entry agreements for innovative and cost-effective therapies might result in a major reduction in HIV care costs. Additionally, the establishment of a coordinated approach can help ensure that the quality of services and care being delivered are standardized and maintained on a national level. This can be achieved by establishing a national HIV disease registry to facilitate data collection; use of unified and approved key performance indicators; and development of recommendations for screening and/or prevention of chronic-diseases and opportunistic infections in HIV patients. The results of combining all these strategies could be promising in terms of improving the quality of care and cost-savings; however, further data on the possible impact of such strategies and interventions are still needed.

One strength of this study is that the data collected were from 2015–2018, so the trend over time was examined. The use of a bottom-up micro-costing approach is considered as another strength of this study, since it generates a more precise estimate compared with a top-down approach, given the fact that the bottom-up approach is more difficult to perform. Another strength of this study is that we covered all the services and therapies that were offered for HIV patients in our hospital. As in all research, our study has certain limitations. First, it is a retrospective, observational, single-center study, and the number of patients is limited, and hence cannot be considered representative of a wider sample because the clinical data were only collected from one center, and therefore, results from this study cannot be generalized for the Saudi population. Second, it was challenging to compare the results of the present study with the findings from other countries due to considerable differences in the design of the studies, service costs, and the resultant population samples. Hence, a comparison of expenditure data of different systems may lead to indistinct inferences. Missing and incomplete data was a third limitation for this study; however, we overcame it by double-checking all extracted patients’ data twice with the help of two researchers, and filling in the gaps in missing or incomplete data by manually reviewing patients’ medical files.

## 5. Conclusions

The total costs of HIV-related healthcare in a tertiary care hospital in Saudi Arabia continue to be high, and vary greatly depending on the patient’s presenting illness, clinical stage, developed opportunistic infection, co-morbidity, and pharmacological therapy. The majority of costs are attributable to ARTs. The study provides significant information on healthcare resource utilization for HIV-infected patients in Saudi Arabia as it can be used to guide policy, planning, implementation, and conducting cost-effectiveness studies for new medications, technologies, and models of care. Moreover, introducing a successful policy, aiming at reducing the number of new infections, early initiation of ARTs, and improving the health status of PLHIV can mitigate the burden of HIV on the economy as a whole, and thus soften the impact on healthcare services and the government budget. Additional research, with the involvement of different centers and the use of patient-specific data, is recommended to consolidate the findings of this analysis.

## Figures and Tables

**Table 1:** Patients’ sociodemographics.

***PLHIV Socio-Demographic***		
Mean age ± (SD)	38.76 ± 11.47	
Mean age at diagnosis ± (SD)	33.73 ± 11.63	
Mean disease duration ± (SD)	5.27 ± 4.81	
Patients socio-demographics	N	%
***Gender***		
Female	6	14.3
Male	36	85.7
***Cumulative number of patients included per year***		
2015	29	69
2016	34	81
2017	40	95
2018	42	100
***Nationality***		
Saudi	37	88.1
Non-Saudi	5	11.9
***Marital status***		
Single	15	35.6
Married	26	62.4
Unknown	1	2.4
***Employment status***		
Non-Employed	4	9.5
Employed	21	50
Unknown	17	40.5
***Has children***		
Yes	16	38.1
No	16	38.1
Unknown	10	23.8

**Table 2:** Patients’ clinical characteristics and treatment regimens.

*CD4 Count (Cells/µL)*	N	%
>500	25	59.5
351–500	9	21.4
200–350	2	4.8
Unmeasured	6	14.3
***Viral load (copies/mL)***		
≥500	7	17
200–499	2	5
50–199	1	2
Undetectable viral load (<50)	26	62
Not available	6	14
***HIV mode of transmission***		
Sexual	11	26.2
Blood transfusion	1	2.4
Needle stick	1	2.4
Undisclosed	29	69
***Co-morbidities***		
Yes	15	35.7
No	27	64.3
***Mortality***		
Died in hospital	1	2.4
***Developed opportunistic infection (N = 21) total number of infections 37***		
Candidiasis	3	8.1
Cryptococcosis	1	2.7
Herpes simplex	3	8.1
Mycobacterium avium complex	1	2.7
Tuberculosis	2	5.4
Pneumocystis jirovecii pneumonia	7	18.9
Pneumonia	4	10.8
CNS Toxoplasmosis	3	8.1
Others	13	35.1
***Regimen types and switching***		
Initiated on STR: Elvitegravir/Cobicistat/Emtricitabine/Tenofovir Disoproxil Fumarate Efavirenz/Tenofovir Disoproxil Fumarate/Emtricitabine	26	61.9
Initiated on MTR Zidovudine/Lamivudine + Lopinavir/Ritonavir	12	28.6
Continued on STR: Efavirenz/Tenofovir Disoproxil Fumarate/Emtricitabine	24	57.1
Continued on MTR: Zidovudine/Lamivudine + Lopinavir/Ritonavir	3	7.1
Switched from initial therapy	10	23.8
Non-switching	27	64.3

**Table 3:** Total cost and percentage impact per year of the cost categories from 2015 to 2018.

	Cost (US$)					% of Total Patient Cost			
	2015	2016	2017	2018	Total	2015	2016	2017	2018
**ARTs**	125,494.94	294,346.22	352,819.16	390,828.99	1,163,489.31	62.56	64.69	52.75	81.44
**Admissions**	44,374.40	69,731.20	198,625.07	41,204.80	353,935.47	22.12	15.32	29.70	8.59
**Laboratory**	22,689.60	71,075.47	95,043.60	34,273.80	223,082.47	11.31	15.62	14.21	7.14
**Outpatient visits**	5704.00	13,805.33	11,986.67	9672.00	41,168.00	2.84	3.03	1.79	2.02
**Radiology**	2332.80	6074.67	10,377.60	3930.13	22,715.20	1.16	1.33	1.55	0.82
**Total**	**200,595.74**	**455,032.89**	**668,852.10**	**479,909.72**	**1,804,390.45**	**100**	**100**	**100**	**100**

**Table 4:** Healthcare resources and medication costs (US$) according to PLHIV characteristics.

		Nationality		Gender		Co-Morbidities		Opportunistic Infection	
Cost		Non-Saudi (*n* = 5)	Saudi (*n* = 37)	Female (*n* = 6)	Male (*n* = 36)	No (*n* = 27)	Yes (*n* = 15)	No (*n* = 21)	Yes (*n* = 21)
**Visits**	**Total**	6944.00	34,224	10,333.33	30,834.67	20,088.00	21,080.00	19,674.67	21,493.33
	**Mean**	1388.80	925	1722.22	856.52	744.00	1405.33	936.89	1023.49
	**Median**	992	826.7	1694.7	868	744	992	909.3	909.3
	**Q1**	909.3	496	744	537.3	413.3	826.7	661.3	578.7
	**Q3**	1488	1157.3	2893.3	1074.7	909.3	2480	992	1157
	***p*-value**	0.15		0.14		**0.022**		0.91	
**Radiology**	**Total**	3909.33	18,805.87	4517.33	18,197.87	6944.00	15,771.20	543.07	17,282.13
	**Mean**	781.87	508.27	752.89	505.50	257.19	1051.41	25.8604762	822.96
	**Median**	224	96	144	110.4	48	288	48	240
	**Q1**	144	0	0	0	0	96	0	0
	**Q3**	240	320	928	313.6	224	2128	208	928
	***p*-value**	0.42		0.98		**0.011**		0.084	
**Laboratory**	**Total**	38,260.27	184,822.21	35,290.67	187,791.81	98,135.55	124,946.93	73,062.67	150,019.81
	**Mean**	7652.05	4995.19	5881.78	5216.44	3634.65	8329.80	3479.17	7143.80
	**Median**	4789.3	3360.3	3553.6	3599.5	3302.4	5223.2	2983	4789
	**Q1**	3884.3	2132	2138.1	2303.2	2132	2710.9	1433	3360
	**Q3**	6418.1	5573.9	6597.1	5884	4519.5	15,541.9	3747	6597
	***p*-value**	0.068		0.93		0.083		**0.013**	
**ARTs**	**Total**	105,371.95	1,058,117.33	192,659.68	970,829.60	713,942.40	449,546.93	549,776.00	613,713.33
	**Mean**	21,074.39	28,597.77	32,109.95	26,967.49	26,442.31	29,969.80	26,179.81	29,224.44
	**Median**	15,840.6	33,892.3	35,924.1	31,616.3	29,864	34,566.6	29,524	34,135
	**Q1**	12,963.3	24,866.5	29,523.5	20,746.4	15,840.6	24,079	13,387	24,079
	**Q3**	26,744.3	37,466.3	37,708.6	37,299.7	38,859.7	37,345.1	36,437	37,466
	***p*-value**	0.11		0.33		0.62		0.46	
**Admissions**	**Total**	14,263.20	339,672.27	30,111.20	323,824.27	88,748.80	265,186.67	47,544.00	306,391.47
	**Mean**	2852.64	9,180.33	5018.53	8995.12	3286.99	17,679.11	2264.00	14,590.07
	**Median**	0	0	3169.6	0	0	6339.2	0	0
	**Q1**	0	0	0	0	0	0	0	0
	**Q3**	0	6339.2	9508.8	4754.4	0	15,848	0	14,263
	***p*-value**	0.55		0.49		**0.001**		0.13	
**Total**	**Total**	168,748.75	1,635,641.60	272,912.27	1,531,478.13	927,858.67	876,531.73	695,490.40	1,108,900.27
	**Mean**	33,749.75	44,206.53	45,485.38	42,541.06	34,365.14	58,435.45	33,118.59	52,804.77
	**Median**	21,448.6	42,911.7	47,791.1	40,587.8	36,472	50,692	37,644	44,731
	**Q1**	17,983.5	34,285.8	29,880.8	29,528.8	21,448.6	44,617.5	18,226	36,472
	**Q3**	34,874.4	50,692	53,007.1	46,599	43,049.9	58,231.9	43,050	58,232
	***p*-value**	0.21		0.42		**0.002**		**0.033**	

## Data Availability

The data presented in this study are available on request from the corresponding author. The data are not publicly available due to ethical restrictions and King Saud University’s College of Medicine Research Ethics Committee prior approval.
